# Local Pain-reducing Methods after Hemorrhoidectomy

**DOI:** 10.1007/s00268-013-1983-z

**Published:** 2013-03-14

**Authors:** Ahmet Okuş

**Affiliations:** Department of General Surgery, Konya Training and Research Hospital, Konya, Turkey

I read with interest the study by Ala and colleagues [1] published in the *World Journal of Surgery* titled “Efficacy of cholestyramine ointment in the reduction of postoperative pain and pain during defecation after open hemorrhoidectomy: results of a prospective, single-center, randomized, double-blind, placebo-controlled trial”. Hemorrhoidal disease is still a common disorder, and one for which surgery is one of the most common treatment methods. Because of the rich innervation of the anal canal, postoperative pain is still the most significant problem, both for the patient and for the surgeon. Postoperative pain requires opiate use, prolongs the hospital stay, and affects the patient’s comfort. In their recent study, Ala et al. used cholestyramine ointment to reduce the pain after hemorrhoidectomy. They aimed to block the irritation and inflammation in the anal canal caused by bile acids found in stool. The use of cholestyramine ointment after hemorrhoidectomy was first investigated in their study [1], in which the authors investigated the efficacy of the compound in reducing postoperative pain at rest and during defecation, as well as the analgesic requirement after open hemorrhoidectomy. Results of the study showed a marked reduction in postoperative pain in the treatment group compared with the control group between postoperative hour 12 and postoperative hour 48 and complete pain elimination by the second postoperative week. In a comparison with placebo, the study also showed a decrease in the requirement for analgesic use after open hemorrhoidectomy.

The toxic and inflammatory effects of bile acids and bile salts are well recognized. These factors cause skin irritation after ileostomy, bile fistula, and ileoanal anastomosis. The preventive effects of cholestyramine ointment on skin irritation caused by bile acids have been demonstrated [2, 3]. Ala et al. emphasized that in their study. However, the conditions after hemorrhoidectomy are fully different from those after ileostomy, bile fistula, or ileoanal anastomosis. Because, more than 95 % of bile acids are absorbed in small bowel and reach the liver via the enterohepatic circulation, only a small proportion can be found in stool. Therefore, it is difficult to say that blockage of this small proportion of bile acids by cholestyramine ointment significantly reduces the pain associated with hemorrhoidectomy. Studies with oral cholestyramine are needed to prove this result.

Moreover, in similar study by Ala and colleagues [4] they used sucralfate ointment after hemorrhoidectomy. Sucralfate is a basic aluminum salt of sucrose octasulfate that is effective in a weakly acidic environment. When applied to the wound, it protects it from mechanical damage and prevents the release of inflammatory cytokines from damaged tissues. Its use was investigated topically for skin excoriation. Like cholestyramine ointment, when compared to placebo, sucralfate ointment was shown to reduce patients’ analgesic use and postoperative pain 12 h after the hemorrhoidectomy. When the two studies are evaluated together, the VAS scores of the placebo groups in each study are expected to be similar. In fact, at 12, 24, and 48 h post-hemorrhoidectomy, the VAS scores of the placebo groups were 3.93 ± 3.85, 4.07 ± 3.35, and 3.57 ± 3.45, respectively. In another study of sucralfate ointment, the VAS scores of the placebo group were 5.16 ± 1.12, 5.08 ± 0.97, and 4.41 ± 0.82 at the 12, 24, and 48 h time points, respectively. This difference should be explained by the authors. In both the sucralfate ointment study and the cholestyramine ointment study, there is no difference in the pain scores at the 12 h time point, confirming the pain-reducing effect of local ointment application. The pain reduction may be related to the local effect of ointment at the first defecation. When the two studies are evaluated together, the common property of the two agents is a pain-reducing affect after hemorrhoidectomy. However, all they have in common is the formation of a barrier that protects the wound. The two studies may trigger the search for new topical agents for reducing pain after hemorrhoidectomy.

## References

[1] Ala S, Eshghi F, Enayatifard R (2013). Efficacy of cholestyramine ointment in reduction of postoperative pain and pain during defecation after open hemorrhoidectomy: results of a prospective, single-center, randomized, double-blind, placebo-controlled trial. World J Surg.

[2] Møller P, Lohmann M, Brynitz S (1987). Cholestyramine ointment in the treatment of perianal skin irritation following ileoanal anastomosis. Dis Colon Rectum.

[3] Rodriguez JT, Huang TL, Ferry GD (1976). Treatment of skin irritation around enterostomies with cholestyramine ointment. J Pediatr.

[4] Ala S, Saeedi M, Eshghi F (2013). Efficacy of 10% sucralfate ointment in the reduction of acute postoperative pain after open hemorrhoidectomy: a prospective, double-blind, randomized, placebo-controlled trial. World J Surg.

